# Supplementary material for: RNA structure probing reveals the structural basis of Dicer binding and cleavage
Source: Nat Commun. 2021 Jun 7;12:3397. doi: 10.1038/s41467-021-23607-w (PMC8184798; doi:10.1038/s41467-021-23607-w)
Supplement: Supplementary file 1 — Supplementary Information [file 41467_2021_23607_MOESM1_ESM.pdf]

Supplementary Information

RNA structure probing reveals the structural basis of Dicer binding and cleavage

Luo et al.

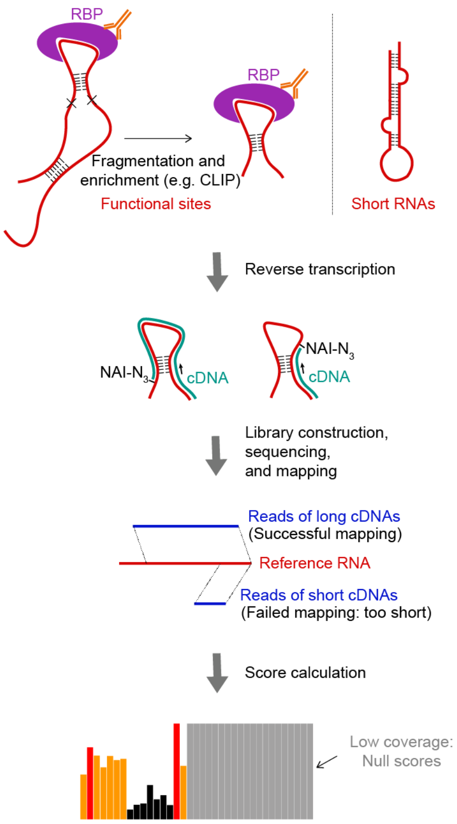

Supplementary Note 1 Structural information at the 3′ terminus of an RNA will be missing in the truncation-based methods of reverse transcription, due to the loss of mapping of short sequencing reads.

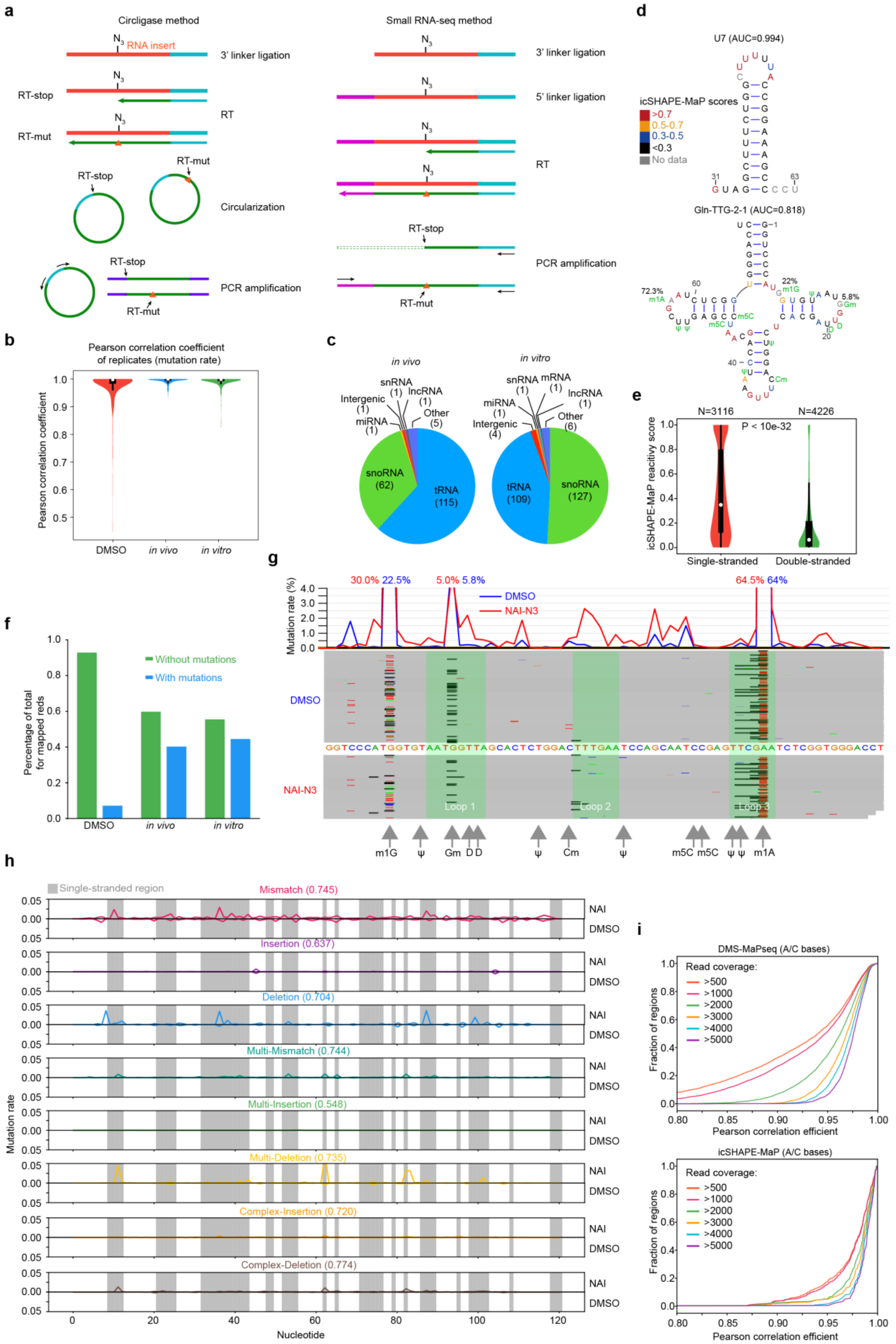
a
Circligase method
Small RNA-seq method
RNA insert
3' linker ligation
5' linker ligation
RT-stop
RT-mut
RT
Circularization
PCR amplification
b
Pearson correlation coefficient of replicates (mutation rate)
Pearson correlation coefficient
DMSO
in vivo
in vitro
c
in vivo
in vitro
snoRNA (62)
tRNA (115)
tRNA (109)
snoRNA (127)
Intergenic (1)
miRNA (1)
snRNA (1)
lncRNA (1)
Other (5)
mRNA (1)
Intergenic (4)
Other (6)
d
U7 (AUC=0.994)
Gln-TTG-2-1 (AUC=0.818)
icSHAPE-MaP scores
>0.7
0.5-0.7
0.3-0.5
<0.3
No data
e
N=3116
N=4226
P < 10e-32
icSHAPE-MaP reactivity score
Single-stranded
Double-stranded
f
Without mutations
With mutations
Percentage of total for mapped reads
DMSO
in vivo
in vitro
g
30.0% 22.5%
5.0% 5.8%
64.5% 64%
Mutation rate (%)
DMSO
NAI-N3
GGTCCCATGGTGTAATGGTTAGCACTCTGGACTTTGAATCCAGCAATCCGAGTTCGAATCTCGGTGGGACCT
Loop 1
Loop 2
Loop 3
m1G
ψ
Gm
D D
ψ
Cm
ψ
m5C m5C
ψ ψ
m1A
h
Single-stranded region
Mismatch (0.745)
Insertion (0.637)
Deletion (0.704)
Multi-Mismatch (0.744)
Multi-Insertion (0.548)
Multi-Deletion (0.735)
Complex-Insertion (0.720)
Complex-Deletion (0.774)
NAI
DMSO
Mutation rate
Nucleotide
i
DMS-MaPseq (A/C bases)
icSHAPE-MaP (A/C bases)
Read coverage:
>500
>1000
>2000
>3000
>4000
>5000
Fraction of regions
Pearson correlation efficient

Supplementary Fig. 1 Profiling structure of small RNAs with a biochemical approach, icSHAPE-MaP. a Two library construction methods are compared. **b** Violin plot of Pearson correlation coefficients for mutation rates among replicates of *in vivo* (red), *in vitro* (blue), or DMSO (green) groups (n=271, 235, or 238, respectively). **c** Pie plot of the number of structure-detected RNAs. **d** Examples of one snoRNA and one tRNA with icSHAPE-MaP scores and their secondary structure models. **e** icSHAPE-MaP score distribution of single-stranded (n=3,116) and double-stranded (n=4,226) nucleotides of tRNAs. *p* values were determined with a two-sided two-sample t test. **f** Ratio of reads with or without mutations of pre-miRNAs and 5S rRNA in three samples. **g** The mutation rate of DMSO and NAI-N_3 samples, and the IGV track of reads mutation for Gln-TTG-2-1 tRNA. The loop regions are highlighted in green. The RNA modifications are collected from the Modomics database, indicated by arrows. m1G: 1-methylguanosine, ψ: pseudouridine, D: dihydrouridine, Gm: 2-O'-methylated guanosine, Cm: 2-O'-methylated cytosine, m5C: 5-methylcytosine, m1A: N^1-methyladenosine. **h** Mutation rate of eight types of mutations in human 5S rRNA. The AUC value for each mutation type is shown. **i** Cumulative distribution curve for Pearson correlation efficient of mutation rate in DMS (upper) or NAI-N3 (lower) samples for A and C bases between two replicates. Each line represents a different read coverage. Violin plots show the median (white circle), 25/75 percentiles, and smallest/largest values within 1.5 times the interquartile range above/below quartiles.

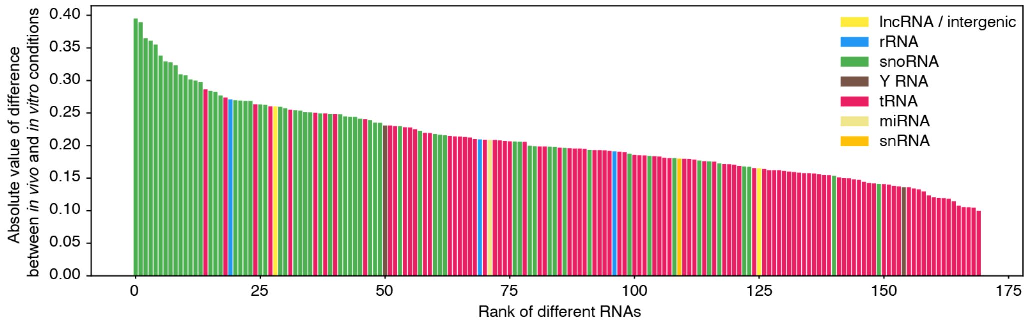

Supplementary Fig. 2 Differential analysis of structure of small RNAs between the *in vivo* and *in vitro* datasets. Structure differences between *in vivo* and *in vitro* icSHAPE-MaP structure profiles, defined as the average absolute difference of SHAPE scores for each nucleotide in each transcript. RNAs are ranked by structure difference and are colored by their type as indicated. Source data are provided as a Source Data file.

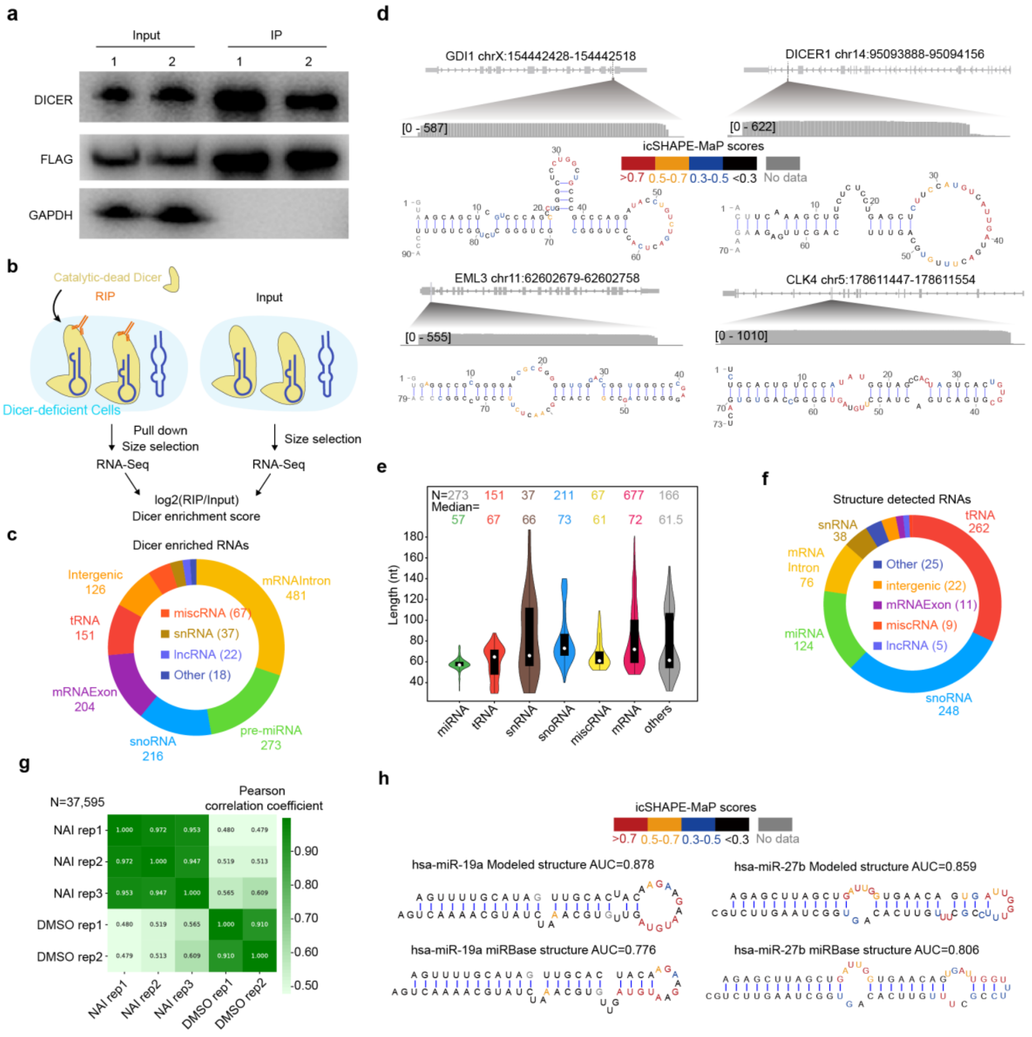

Supplementary Fig. 3 Development of RIP-icSHAPE-MaP for Dicer substrates. a Western blotting for input and pull-down sample (IP) in RIP performed in Dicer-deficient 293T cells overexpressing Flag-tagged catalytic-dead Dicer. The experiment was repeated two times with similar results. Source data are provided as a Source Data file. **b** Cartoon of RNA-Seq (for ~40-200 nt long RNAs) experiment design and calculation of enrichment score. **c** Ring diagram of the number of different types of Dicer substrates. **d** Reads coverage and secondary structures of four fragments from GDI1, DICER1, EML3, or CLK4. **e** Size distribution for different types of Dicer-bound RNA fragments. Violin plots show the median (white circle), 25/75 percentiles, and smallest/largest values within 1.5 times the interquartile range above/below quartiles. **f** Ring diagram of the number of different types of RNAs with icSHAPE-MaP scores. **g** Heatmap of Pearson correlation efficient of mutation rate per nucleotide between two replicates in DMSO or NAI-N_3 samples. **h** The secondary structure models and icSHAPE-MaP scores of hsa-miR-19a and hsa-miR-27b pre-miRNAs. The structures on the top were modeled using the RNAStructure software with their icSHAPE-MaP scores as constraints. The structures on the bottom are from the miRBase database.

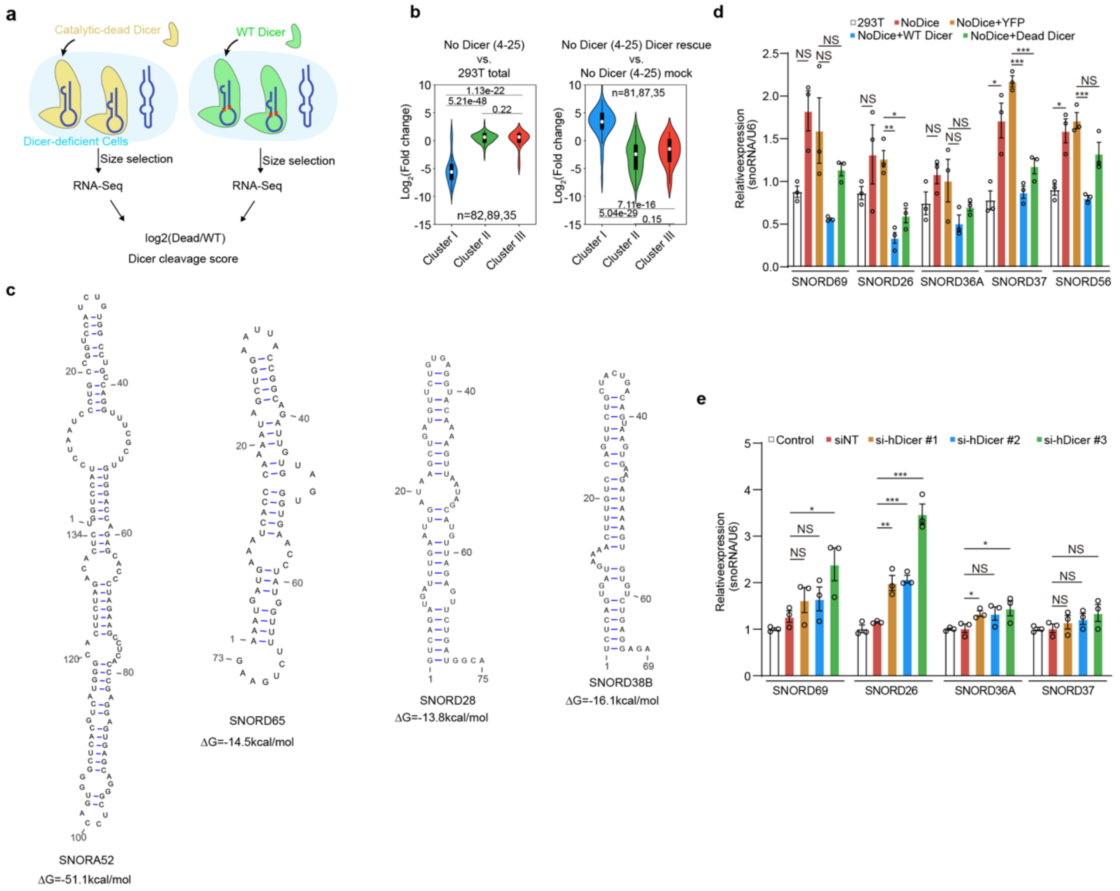

Supplementary Fig. 4 Dicer acts as an endonuclease in RNA metabolism, independent of miRNA biogenesis. a Cartoon of RNA-Seq (for ~40-200 nt long RNAs) experiment design and calculation of cleavage score. **b** Log2 fold change of small RNA (~20-nt) expression level mapping to the three clusters of Dicer substrates between Dicer deficiency (the NoDice4-25 cell line) vs. HEK293T and Dicer rescue vs. mock transfection. sRNA datasets are from Bogerd et al., 2014. **c** Secondary structure Models of four snoRNAs as Dicer cleavage substrates, predicted by *RNAstructure* using their icSHAPE-MaP scores as constraints. **d** qPCR showing the expression level of select snoRNAs with Dicer complementation. “293T”: wild type HEK293T cell line; “NoDice”: Dicer deficient HEK293T cell line; “NoDice+YFP”: Dicer deficient HEK293T cell line overexpressing a plasmid of YFP using the same vector backbone as the groups “NoDice+WT/Dead Dicer”; “NoDice+WT Dicer”: Dicer deficient HEK293T cell line overexpressing a plasmid for wild type Dicer; “NoDice+Dead Dicer”: Dicer deficient HEK293T cell line overexpressing a plasmid for catalytic-dead Dicer. Data are the mean ± SD, n = 3 biological replicates. *p* values were determined with a two-sided two-sample Student’s t-test (*, p<0.05; **, p<0.01; ***, p<0.001; NS, not significant). **e** qPCR showing the expression level of select snoRNAs in 293T cells with Dicer knockdown by siRNAs. “Control”: HEK293T cells treated with Lipofectamine as the transfection reagent; “siNT”: scramble siRNAs. “si-hDicer #1/#2/#3”: three different siRNAs against human Dicer. Data are the mean ± SD, n = 3 biological replicates. *p* values were determined with a two-sided two-sample Student’s t-test (*, p<0.05; **, p<0.01; ***,p<0.001; NS, not significant). Source data are provided as a Source Data file.

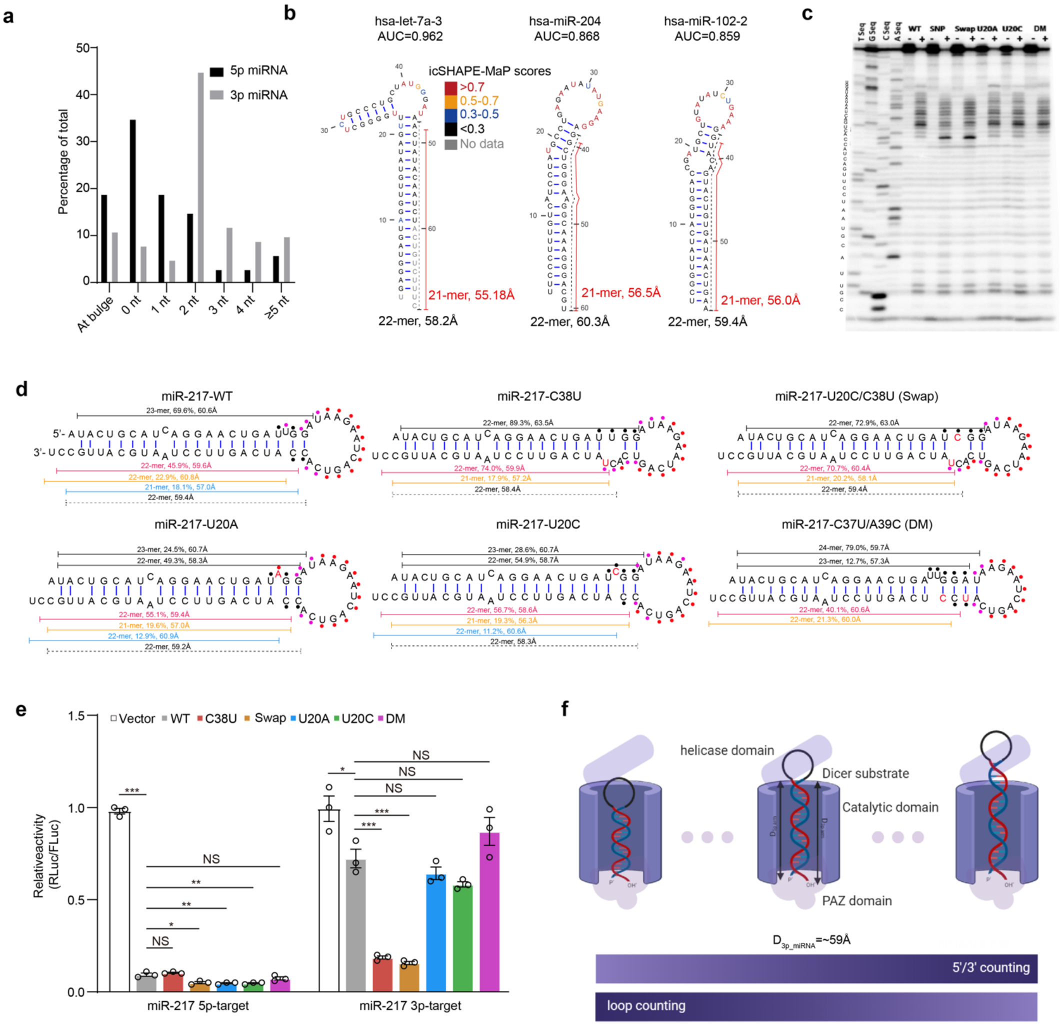
a
b
c
d
e
f
5p miRNA
3p miRNA
Percentage of total
At bulge
0 nt
1 nt
2 nt
3 nt
4 nt
≥5 nt
hsa-let-7a-3
AUC=0.962
hsa-miR-204
AUC=0.868
hsa-miR-102-2
AUC=0.859
icSHAPE-MaP scores
>0.7
0.5-0.7
0.3-0.5
<0.3
No data
21-mer, 55.18Å
22-mer, 58.2Å
21-mer, 56.5Å
22-mer, 60.3Å
21-mer, 56.0Å
22-mer, 59.4Å
T Seq
G Seq
C Seq
A Seq
WT
SNP
Swap
U20A
U20C
DM
miR-217-WT
miR-217-C38U
miR-217-U20C/C38U (Swap)
miR-217-U20A
miR-217-U20C
miR-217-C37U/A39C (DM)
Vector
WT
C38U
Swap
U20A
U20C
DM
Relativeactivity (RLuc/FLuc)
NS
miR-217 5p-target
miR-217 3p-target
helicase domain
Dicer substrate
Catalytic domain
PAZ domain
D_{3p_miRNA}=~59Å
5'/3' counting
loop counting

Supplementary Fig. 5 Dicer measures spatial distance for cleavage-site selection on pre-miRNAs. **a** Distribution of the loop position in secondary structures for human pre-miRNA. Pre-miRNAs are categorized based on the number of nucleotides between the Dicer cleavage site (determined by the 3′ end of 5p miRNA or 5′ end of 3p miRNA) and the nearest upstream bulge or its terminal loop. If Dicer cleaves inside a loop/bulge, this miRNA is regarded as "At bulge". The miRNA structure information was generated using RNAStructure with their icSHAPE-MaP scores as constraints. **b** Select examples of pre-miRNAs for not generating a 22-mer with a closer D_{3p_miRNA} to 59 Å (black dash line), potentially violating the loop counting rule. The annotated miRNAs are shown in red lines. **c** *In vitro* SHAPE analysis for pre-miR-217 variants. **d** Isoforms from pre-miR-217 variants could not be produced, owing to structural constraints or other reasons (see main text), even when they had a D_{3p_miRNA} in proximity to 59 Å. **e** Dual luciferase assays were used to monitor the repression activities of miR-217 isoforms. Renilla luciferase activities were normalized to Firefly luciferase and were plotted as the percentage of relative enzyme activity compared to the negative control (transfected with a plasmid vector expressing mmu-pre-miR-124-1 only). Data are the mean ± SD, n = 3 biological replicates. *p* values were determined with a two-sided two-sample Student's t-test (*, $p<0.05$; **, $p<0.01$; ***, $p<0.001$; NS, not significant). **f** Working model for Dicer cleavage-site selection based on the D_{3p_miRNA}. Pre-miRNA termini locate within the PAZ domain of Dicer with their loops interacting with the helicase domain (middle panel). Dicer preferentially cleaves at D_{3p_miRNA} in proximity to 59.2±1Å, which determines the nucleotide lengths of mature miRNAs. In the scenario of pre-miRNAs with shorter arms (D_{3p_arm}), the loop counting rule or other structural features may dictate the Dicer cleavage-site selection (left panel). For pre-miRNAs with longer arms, the 5′/3′ counting rules may be the primary determinant for Dicer cleavage-site selection (right panel). This panel is created with BioRender.com. Source data are provided as a Source Data file.
