# Supplementary material for: RNA structure probing reveals the structural basis of Dicer binding and cleavage
Source: Nat Commun. 2021 Jun 7;12:3397. doi: 10.1038/s41467-021-23607-w (PMC8184798; doi:10.1038/s41467-021-23607-w)
Supplement: Supplementary file 10 — Description of additional supplementary files [file 41467_2021_23607_MOESM10_ESM.docx]

Description of additional supplementary information

Title: Supplementary Data 1

Description: Comparison of Dicer binding sites identified by RIP (this study) and PAR-CLIP (Rybak-Wolf et al, Cell, 2014).

Title: Supplementary Data 2

Description: The distribution of mapped reads in RIP-icSHAPE-MaP.

Title: Supplementary Data 3

Description: Details about Dicer substrates discovered by RIPicSHAPE-MaP.

Title: Supplementary Data 4

Description: Details about structure modeling and rules of Dicer cleavage-site selection on pre-miRNAs obtained by RIP-icSHAPE-MaP.

Title: Supplementary Data 5

Description: Summary about arm lengths, mature miRNA lengths and different rules of Dicer cleavage-site selection on pre-miRNAs obtained by RIP-icSHAPE-MaP.

Title: Supplementary Data 6

Description: Oligo and probe sequences used in this study, related to Methods.

Title: Supplementary Data 7

Description: The read number mapped to each pre-miRNA identified in the RIP- icSHAPE-MaP data of this study.
