# Supplementary material for: RNA structure probing reveals the structural basis of Dicer binding and cleavage
Source: Nat Commun. 2021 Jun 7;12:3397. doi: 10.1038/s41467-021-23607-w (PMC8184798; doi:10.1038/s41467-021-23607-w)
Supplement: Supplementary file 11 — Reporting summary [file 41467_2021_23607_MOESM11_ESM.pdf]

nature research

Corresponding author(s): Qiangfeng Cliff Zhang, Mark A. Kay

Last updated by author(s): Apr 22, 2021

Reporting Summary

Nature Research wishes to improve the reproducibility of the work that we publish. This form provides structure for consistency and transparency in reporting. For further information on Nature Research policies, see our Editorial Policies and the Editorial Policy Checklist.

Statistics

For all statistical analyses, confirm that the following items are present in the figure legend, table legend, main text, or Methods section.

n/a	Confirmed	
☐	☒	The exact sample size (*n*) for each experimental group/condition, given as a discrete number and unit of measurement
☐	☒	A statement on whether measurements were taken from distinct samples or whether the same sample was measured repeatedly
☐	☒	The statistical test(s) used AND whether they are one- or two-sided<br>*Only common tests should be described solely by name; describe more complex techniques in the Methods section.*
☒	☐	A description of all covariates tested
☐	☒	A description of any assumptions or corrections, such as tests of normality and adjustment for multiple comparisons
☐	☒	A full description of the statistical parameters including central tendency (e.g. means) or other basic estimates (e.g. regression coefficient) AND variation (e.g. standard deviation) or associated estimates of uncertainty (e.g. confidence intervals)
☐	☒	For null hypothesis testing, the test statistic (e.g. *F*, *t*, *r*) with confidence intervals, effect sizes, degrees of freedom and *P* value noted<br>*Give P values as exact values whenever suitable.*
☒	☐	For Bayesian analysis, information on the choice of priors and Markov chain Monte Carlo settings
☒	☐	For hierarchical and complex designs, identification of the appropriate level for tests and full reporting of outcomes
☐	☒	Estimates of effect sizes (e.g. Cohen's *d*, Pearson's *r*), indicating how they were calculated

Our web collection on statistics for biologists contains articles on many of the points above.

Software and code

Policy information about availability of computer code

Data collection	The collection of Illumina sequencing data was described in the "Methods" section.<br>RNA-seq: Illumina NextSeq 500, HiSeq X Ten, and MiSeq.<br>Image J (version 1.49) was used to quantify the darkness of bands in gels.
Data analysis	The sequencing data was processed by removing adapters with cutadapt (Version 1.16), filtering high-quality reads with Trimmomatic (Version 0.33) and duplication removal with an in-house Perl script. Clean reads were mapped to the reference sequences with STAR (Version 2.7.1a). Replicate samples were combined with samtools (Version 1.3.1), and Shapemapper2 (Version 2.1.4) was used to calculate final scores. The Fold program in the RNAstructure package (Version 5.6) was used to predict the secondary structures of RNAs. RNA secondary structures were visualized with the VARNAv3-93 command line. The PCA analysis was carried out with the Sklearn (Version 0.20.3) package. De novo tertiary structure models of pre-miRNAs were generated by Rosetta (Version 2019.12.60667). The Python (v3.7) source code for the analysis is available on Github [https://github.com/lipan6461188/RIP-icSHAPE-MaP] or [http://doi.org/10.5281/zenodo.4680657]

For manuscripts utilizing custom algorithms or software that are central to the research but not yet described in published literature, software must be made available to editors and reviewers. We strongly encourage code deposition in a community repository (e.g. GitHub). See the Nature Research guidelines for submitting code & software for further information.

Data

Policy information about availability of data

All manuscripts must include a data availability statement. This statement should provide the following information, where applicable:

- Accession codes, unique identifiers, or web links for publicly available datasets
- A list of figures that have associated raw data
- A description of any restrictions on data availability

We have used publicly available datasets of RNA modifications (http://genesilico.pl/modomics/), miRNA sequences and secondary structure models (http://www.mirbase.org/), and tRNA sequences and secondary structure models (http://gtrnadb.ucsc.edu/), snoRNA and snRNA sequences (https://www.gencodegenes.org/) and vaultRNA and YRNA sequences (https://www.ncbi.nlm.nih.gov/refseq/). The source data for Figs. 1-4, as well as Supplementary Figs. 2-5, is provided as a Source Data file. icSHAPE-MaP and RNA-Seq data that support the findings of this study have been deposited in GEO with the primary accession codes GSE146952 (https://www.ncbi.nlm.nih.gov/geo/query/acc.cgi?acc=GSE146952)

Field-specific reporting

Please select the one below that is the best fit for your research. If you are not sure, read the appropriate sections before making your selection.

☒ Life sciences ☐ Behavioural & social sciences ☐ Ecological, evolutionary & environmental sciences

For a reference copy of the document with all sections, see nature.com/documents/nr-reporting-summary-flat.pdf

Life sciences study design

All studies must disclose on these points even when the disclosure is negative.

Sample size	No sample size calculation was performed. The sample size of experiments was determined by the following related studies on structure probing, including icSHAPE (Spitale et al. Structural imprints in vivo decode RNA regulatory mechanisms. Nature.2015) and DMSMaP-seq (Zubradt et al. DMS-MaPseq for genome-wide or targeted RNA structure probing in vivo. Nature Method.2016), and studies on Dicer substrates (Rybak-Wolf et al. A Variety of Dicer Substrates in Human and C. elegans. Cell. 2014), and studies on Dicer cleavage using sRNA-seq (Gu et al. The Loop Position of shRNAs and Pre-miRNAs Is Critical for the Accuracy of Dicer Processing In Vivo. Cell. 2012). And the samples sizes used in this study are acceptable and adequate for statistical tests.
Data exclusions	No data were excluded.
Replication	All experiments contained two to four biological replicates. And all replicates were reproducible with similar results.
Randomization	The cells and samples were randomly assigned to treated or non-treated groups for this study.
Blinding	Investigators were not blinded to the group allocation since the investigators need to perform different treatments when collecting data.

Reporting for specific materials, systems and methods

We require information from authors about some types of materials, experimental systems and methods used in many studies. Here, indicate whether each material, system or method listed is relevant to your study. If you are not sure if a list item applies to your research, read the appropriate section before selecting a response.

Materials & experimental systems

n/a	Involved in the study
☐	☒ Antibodies
☐	☒ Eukaryotic cell lines
☒	☐ Palaeontology and archaeology
☒	☐ Animals and other organisms
☒	☐ Human research participants
☒	☐ Clinical data
☒	☐ Dual use research of concern

Methods

n/a	Involved in the study
☒	☐ ChIP-seq
☒	☐ Flow cytometry
☒	☐ MRI-based neuroimaging

Antibodies

Antibodies used	The antibodies used for Western blots are listed as follows. Anti-GAPDH, rabbit monoclonal, Abcam cat. #ab181602, diluted 1:10,000; Anti-FLAG, mouse monoclonal, Sigma-Aldrich, cat. #F1804, diluted 1:1,000; Anti-DICER, rabbit polyclonal, Proteintech cat. #20567-1-AP, diluted 1:500. Goat anti-mouse IgG (H + L)-HRP Conjugate (EASYBIO cat. #BE0102), diluted 1:2,000; Goat anti-rabbit IgG (H + L)-HRP conjugate (EASYBIO cat. #BE0101), diluted 1:2,000.

Validation	anti-GAPDH: Chen L et al. Long noncoding RNA SOX2OT promotes the proliferation of pancreatic cancer by binding to FUS. Int J Cancer 147:175-188 (2020);<br>anti-FLAG: Monika Srivastava et al. Roquin binds microRNA-146a and Argonaute2 to regulate microRNA homeostasis. Nature communications, 6, 6253-6253 (2015-02-24);<br>anti-DICER: Yan-Su Chen et al. Dicer suppresses MMP-2-mediated invasion and VEGFA-induced angiogenesis and serves as a promising prognostic biomarker in human clear cell renal cell carcinoma. Oncotarget (2016);<br>Goat anti-mouse IgG (H + L)-HRP Conjugate: Jingwen Si et al. Hematopoietic Progenitor Kinase1 (HPK1) Mediates T Cell Dysfunction and Is a Druggable Target for T Cell-Based Immunotherapies. Cancer Cell(2020);<br>Goat anti-rabbit IgG (H + L)-HRP conjugate: Jingwen Si et al. Hematopoietic Progenitor Kinase1 (HPK1) Mediates T Cell Dysfunction and Is a Druggable Target for T Cell-Based Immunotherapies. Cancer Cell(2020);

Eukaryotic cell lines

Policy information about cell lines

Cell line source(s)	293T cells were purchased from ATCC cat. #CRL-3216.<br>293T with Dicer deficiency (NoDice 2-20) was a gift from Dr. Bryan R. Cullen at Duke University.
Authentication	293T cells with Dicer deficiency (NoDice 2-20) were validated by western blot to confirm the absence of full length Dicer and by small RNA blots to confirm the loss of select mature microRNAs. 293T cells was not authenticated but verified by morphological characteristics.
Mycoplasma contamination	All cell lines were tested negative for mycoplasma contamination.
Commonly misidentified lines<br>(See ICLAC register)	None
